# Physical Activity Improves Quality of Life in Patients With Inflammatory Bowel Disease: A Systematic Review and Meta‐Analysis

**DOI:** 10.1002/jcsm.70206

**Published:** 2026-02-01

**Authors:** Emese Kasznár, Barbara Csendes, Dorottya Gergő, Péter Hegyi, Réka Tóth, András Fogarasi, Miklós Garami, Klementina Ocskay, Andrea Párniczky, Mahmoud Obeidat, Katalin Eszter Müller

**Affiliations:** ^1^ Heim Pál National Pediatric Institute Budapest Hungary; ^2^ Centre for Translational Medicine Semmelweis University Budapest Hungary; ^3^ Institute of Genomic Medicine and Rare Disorders Semmelweis University Budapest Hungary; ^4^ Department of Pharmacognosy Semmelweis University Budapest Hungary; ^5^ Institute for Translational Medicine, Medical School University of Pécs Pécs Hungary; ^6^ Institute of Pancreatic Diseases Semmelweis University Budapest Hungary; ^7^ Bethesda Children's Hospital Budapest Hungary; ^8^ András Pető Faculty Semmelweis University Budapest Hungary; ^9^ Pediatric Center Semmelweis University Budapest Hungary; ^10^ Pharmaceutical Sciences and Health Technologies Division, Doctoral School Semmelweis University Budapest Hungary; ^11^ Department of Family Care Methodology, Faculty of Health Sciences Semmelweis University Budapest Hungary

**Keywords:** aerobic fitness, disease activity, inflammatory bowel disease, muscle strength, physical activity, quality of life, sarcopenia

## Abstract

**Background:**

Patients with inflammatory bowel disease (IBD) are often less active physically and experience impaired bone mineral density (BMD) as well as sarcopenia, which are associated with a higher risk of poor disease outcomes. Physical exercise can improve BMD and sarcopenia and may also play a role in controlling inflammation via anti‐inflammatory myokines. This study aimed to evaluate the effects of structured physical exercise on the disease activity and quality of life of patients with IBD by conducting a before‐and‐after analysis.

**Methods:**

We conducted a systematic search on 16 November 2023. Studies with structured exercise interventions in patients with IBD focusing on quality of life, disease activity, body composition, muscle strength, inflammatory markers, aerobic fitness and sedentary time were included. The mean difference (MD) or the standardized MD (SMD) of the differences between the before and after values was used to measure the effect size with a 95% confidence interval (CI). Risk of bias was assessed using the MINORS tool.

**Results:**

Twenty‐one studies involving 498 patients with IBD were included. Quality of life improved significantly: SMD 0.55 (CI, 0.30–0.80), particularly in the systemic subscale. Disease activity scores showed a non‐significant improvement: SMD −0.20 (CI, −0.47 to 0.07) in Crohn's disease and SMD −0.23 (CI, −0.46 to 0.00) in ulcerative colitis. Numbers of inflammatory markers and faecal calprotectin levels also improved, but not significantly. Muscle strength and aerobic fitness improved, with a significant increase in VO_2_ max: SMD 1.88 (CI, 1.34–2.43). Due to the lack of available data, we were unable to conduct a statistical analysis of body composition parameters and sedentary time.

**Conclusion:**

Regular physical activity improves quality of life and physical fitness of IBD patients, with potential benefits for disease activity and sarcopenia.

**Abbreviations**
BFM: body fat mass
BMD: bone mineral density
CD: Crohn's disease
CDAI: Crohn's disease activity index
CI: confidence interval
CRP: C‐reactive protein
ESR: erythrocyte sedimentation rate
HRQoL: health‐related quality of life
IBD: inflammatory bowel disease
LBM: lean body mass
MD: mean difference
MINORS: methodological index for non‐randomized studies
PCDAI: paediatric Crohn's disease activity index
PUCAI: paediatric ulcerative colitis activity index
QoL: quality of life
SMD: standardized mean difference
SMM: skeletal muscle mass
UC: ulcerative colitis
VO_2_ peak: maximal oxygen uptake

## 1 Introduction

The prevalence of inflammatory bowel disease (IBD) represents a significant public health concern, characterized by a rising number of patients with increased risk for mortality and disability‐adjusted life‐years [1]. In 2019, approximately 4.9 million individuals worldwide were diagnosed with IBD, and 147 out of 204 countries or territories reported an increase in the age‐standardized prevalence rate [1]. Approximately 15%–25% of patients are diagnosed with IBD during childhood [2], and as IBD is an incurable, lifelong disease, it is essential to reduce the disease burden without escalating medication use.

Patients with IBD tend to exhibit reduced levels of physical activity as a consequence of their symptoms. Some data indicate that they live a more sedentary life even when in remission compared with healthy people [3, 4]. Their disease and lack of physical activity lead to decreased bone mineral density (BMD), muscle mass and function [5, 6]. A recent review has demonstrated that sarcopenia in patients with IBD is significantly associated with an increased risk of therapy failure, postoperative complications and low BMD [7].

It is important to recognize that IBD affects both the body and the mind, both of which play an important role in quality of life (QoL). Health‐related QoL (HRQoL) is a multi‐dimensional concept and a useful indicator of overall health, as it provides information on an individual's physical and mental health status, social relationships and personal beliefs. There is a bidirectional association between IBD and mental health status, and both of them have a critical impact on QoL [8]. A review published in 2021 found that both depression and anxiety tend to influence disease activity, trigger flares and increase healthcare utilization in patients with IBD [9].

Similarly, physical activity also affects the body and the mind. It is known to improve mood and reduce stress in healthy individuals, contributing to better QoL, and its benefits are being increasingly explored in the context of chronic diseases [10, 11].

In a previous systematic review and meta‐analysis on this topic, the authors found that physical activity can have beneficial effects on reducing disease activity and improving QoL when they compared adult patients with IBD and healthy controls with structured exercise interventions for at least 4 weeks [10]. However, it remains unclear whether this improvement is clinically meaningful and how it affects children. The objective of this study was to conduct a before‐and‐after analysis to evaluate the effects of structured physical exercise on patients with IBD, irrespective of age or disease activity.

## 2 Methods

The systematic review and meta‐analysis followed the Cochrane Handbook [12] and adhered to the Preferred Reporting Items for Systematic Reviews and Meta‐Analyses (PRISMA) 2020 statement [13] (Table S1). The study protocol was registered in advance on PROSPERO (CRD42023483457), and we adhered to it.

### 2.1 Eligibility Criteria

The study population consisted of both paediatric and adult patients with IBD who received structured exercise interventions in addition to standard care. The exercise interventions included physical activities such as walking, running, cycling, resistance and endurance training, yoga and exergames. We did not exclude trials that included dietary advice or supplements in addition to exercise. The primary outcomes were QoL and disease activity scores. Secondary outcomes were body composition: BMD, lean body mass/fat‐free mass (LBM), skeletal muscle mass (SMM) and body fat mass (BFM); muscle strength: handgrip strength (HGS), planking and isometric muscle strength; inflammatory markers: faecal calprotectin, C‐reactive protein (CRP) and erythrocyte sedimentation rate (ESR); aerobic fitness: maximal oxygen uptake (VO_2_ peak); and sedentary time. The study included randomized controlled trials (RCTs), non‐randomized trials, cohort studies, case–control studies, pilot studies, conference abstracts and case series. However, case reports were excluded to maintain objectivity and to avoid subjective evaluations.

### 2.2 Information Sources

On 16 November 2023, a systematic search was conducted on three databases: EMBASE, PubMed and Cochrane Central Register of Controlled Trials (CENTRAL). In addition, a backward and forward citation search was performed using a reference‐checking tool to identify all potential references [14].

### 2.3 Search Strategy

The search key comprised two domains: IBD and physical interventions. No limits were set for the publication period, and no language or other restrictions were applied during the search (Table S2).

### 2.4 Selection Process

The studies were imported into the reference management software, EndNote 20 [15]. After the automatic and manual removal of duplicates, two independent authors conducted title–abstract selection (E.K. and B.C.) and subsequently screened the full texts. Disagreements were resolved after a discussion with a third author (D.G.). The Cohen's kappa coefficient was calculated after each selection phase.

### 2.5 Data Collection Process

Data from eligible studies were extracted by two independent researchers (E.K. and B.C.) using an Excel data collection form Office 365 by Microsoft [16]. Any questions or discrepancies related to data extraction were resolved through discussion.

### 2.6 Data Items

Data extracted from the studies included first author, year of publication, digital object identifier, study design, study period, country, number of participating centres, total number of patients, age of the population, sex distribution, IBD type, disease activity status, exercise intervention, length of intervention, frequency of the intervention, time of measurements, QoL and disease activity scores, BMD, LBM or FFM, SMM, BFM, HGS, planking, isometric muscle strength, faecal calprotectin, CRP, ESR, VO_2_ peak and sedentary time.

### 2.7 Study Risk of Bias Assessment

The risk of bias assessment for each study was independently performed by two investigators (E.K. and B.C.) using the methodological index for non‐randomized studies (MINORS) tool [17]. Disagreements were resolved through discussion. The study evaluated the eight items of the MINORS and conducted an overall bias assessment based on (1) a clearly stated aim, (2) inclusion of consecutive patients, (3) prospective data collection, (4) appropriate endpoints, (5) unbiased assessment of the study endpoint, (6) appropriate follow‐up period, (7) loss to follow‐up less than 5% and (8) prospective calculation of study size.

### 2.8 Synthesis Methods

All statistical analyses were conducted using R [18] with the meta [19] package, using random‐effects models. The mean of the differences between the measurements before and after the intervention was used as the effect size measure. Sample size, mean and corresponding standard deviation (SD) of the measurements before and after the intervention were extracted (separately by the reported subgroup). Standard errors (SE), confidence intervals (CI), medians, interquartile ranges (IQR), minima and maxima were also extracted. If the same data were directly available for the difference, they were also extracted, as well as *t* or *p* values from the paired *t*‐tests. To calculate the SDs from the CI or *p* values, we assumed a t distribution. To estimate means and SDs from medians and IQR and/or range, we used the Luo [20] and Shi [21] methods, as implemented in the meta package.

MDs were used when studies assessed the same outcome with the same measurement units, allowing direct comparison of raw score changes. For studies using different instruments to assess the same construct (e.g., HRQoL questionnaires and disease activity indices), SMDs were calculated by dividing the mean change score (post‐intervention minus baseline) by the baseline SD, as recommended by Harrer et al. [22] to standardize effect sizes across scales. We also clarified that pooled estimates were calculated using inverse‐variance weighting under a random‐effects model, and heterogeneity was quantified using *τ* ^2^ from the restricted maximum‐likelihood estimator with the Q‐profile method for CIs [22, 23].

We estimated the correlation coefficient between the before and after values based on the extracted, calculated or estimated SD of the change when the SD for the baseline and after‐treatment value was also available [12]. If this resulted in a value above one or a negative value, we did not use it. Without information on the SD of the change, the change in the SD was calculated for all studies that used the average of the remaining correlation coefficients for the given outcome. If no other correlation coefficients were available, a value of 0.6 was assumed. The sensitivity analysis used coefficients between 0.1 and 0.9 instead of the average or the default, leading to almost identical results.

We used a Hartung–Knapp adjustment [24, 25] for the CIs. For subgroup analysis, we used a fixed‐effects ‘plural’ model (aka mixed‐effects model). To assess the difference between the subgroups, we used a ‘Cochrane Q’ test (an omnibus test) between subgroups [22]. We summarized meta‐analysis findings in forest plots.

In order to guarantee the accuracy and reliability of the synthesis, every study that provided sufficient data was included. Conversely, studies that lacked sufficient data were excluded.

We planned subgroup analysis for each outcome based on disease type (CD vs. UC), age group (children vs. adults) and disease activity (active disease vs. remission).

## 3 Results

### 3.1 Search and Selection

**FIGURE 1:**
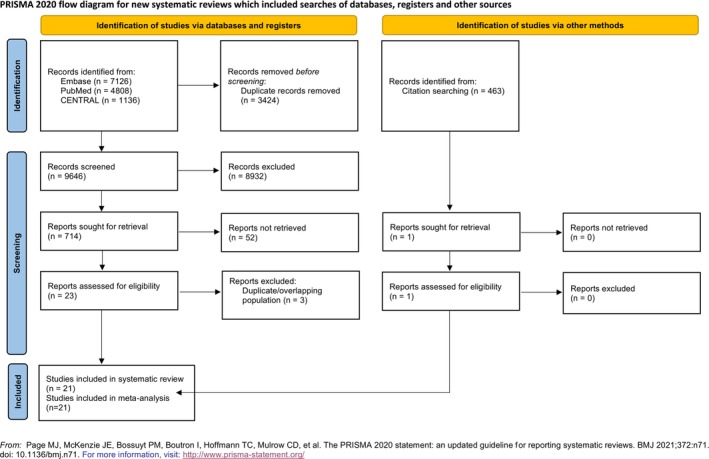
PRISMA 2020 flowchart representing the study selection process.

A total of 13 070 records were identified in the systematic search. After title and abstract selection, we found 714 eligible studies during the full‐text selection, of which 21 were included in the quantitative analysis (Figure 1).

### 3.2 Basic Characteristics of Studies Included

The characteristics of the studies included are summarized in Table 1. Of the 21 included studies, 10 were RCTs [26, 27, 28, 29, 30, 31, 32, 33, 34, 35] and 11 were uncontrolled before–after studies [36, 37, 38, 39, 40, 41, 42, 43, 44, 45, 46]. The studies involved 498 patients with IBD (56% female). Twelve studies included patients with both Crohn's disease (CD) and ulcerative colitis (UC) [27, 29, 32, 36, 37, 38, 39, 40, 41, 42, 44, 45], seven included only patients with CD [28, 30, 31, 33, 34, 43, 46], one included only patients with UC [26] and one did not specify the disease type [35]. Participants were described as in remission, with inactive to mildly active disease and mild to moderately active disease, and three studies did not specify disease activity [38, 39, 46]. Six studies were conducted in a paediatric population with a minimum age of 6 years and maximum age of 21 years [32, 36, 37, 41, 42, 44]; and 15 in the adult population [26, 27, 28, 29, 30, 31, 33, 34, 35, 38, 39, 40, 43, 45, 46].

**TABLE 1:** Characteristics of studies included in the meta‐analysis and systematic review.

Study (first author, year)	Country	Study design	Age group	Participants (n)	Disease types	Disease status	Intervention	Length of intervention	Outcome measures
Arruda, 2018	USA	Prospective, non‐randomized pilot study	Paediatric	9	CD and UC	Remission	Yoga	8 weeks, 3–4 times/week for 30–60 min	Disease activity index, faecal calprotectin
Bjelica, 2023	Canada	Pilot study	Paediatric	10	CD and UC	Remission	Aerobic and resistance training + high protein beverage after every session	16 weeks, 3 times/week for 30–60 min	BMD, BFM, LBM, HGS, VO_2_ peak, sedentary time
Legeret, 2019	Switzerland	Case–control intervention study	Paediatric	21	CD, UC and IBD‐U	Active and remission	Exergames	8 weeks, 5 times/week for 30 min	Disease activity index, CRP, ESR
Mählmann, 2017	Switzerland	Case–control intervention study	Paediatric	21	CD and UC	Active and remission	Exergames	9 weeks, 5 times/week for 30 min	Sedentary time
Scheffers, 2023	The Netherlands	Prospective single‐center randomized semi‐crossover controlled trial	Paediatric	15	CD and UC	Active and remission	Personalized aerob and resistance training + personalized healthy dietary advice	12 weeks, 2–3 times/week for 60 min	QoL, disease activity index, faecal calprotectin, CRP, ESR, BFM, HGS, sedentary time
Trivić, 2023	Croatia	Pre–post interventional study	Paediatric	42	CD, UC and IBD‐U	Remission	Personalized, home‐based structured exercise programme	26 weeks, 3 times/week	Faecal calprotectin, CRP, BMD, BFM, LBM, sedentary time
Cramer, 2017	Germany	Single‐blind randomized controlled trial	Adult	39	UC	Remission	Yoga	12 weeks, 1/week for 90 min	QoL, disease activity score, faecal calprotectin, CRP, ESR
Cronin, 2019	Ireland	Randomized, partial cross‐over trial	Adult	13	CD and UC	Remission	Aerobic and resistance training	8 weeks	QoL, disease activity score, CRP, LBM, BFM, VO_2_ peak
Jones, 2020	UK	Two‐arm, randomized, parallel‐group and assessor‐blind trial	Adult	23	CD	Remission	Impact and high‐effort resistance exercises	26 weeks, 3 times/week for 60 min	QoL, BMD, HGS
Kaur, 2021	Canada	Pre‐ and post‐intervention pilot study	Adult	9	CD and UC	Active and remission	Yoga + meditation, breathing and lavender oil foot massage	8 weeks, 7 times/week for 30 min	QoL, disease activity score
Klare, 2015	Germany	Prospective randomized controlled trial	Adult	15	CD and UC	Remission	Running	10 weeks, 3 times/week	QoL, disease activity score, faecal calprotectin, CRP
Loudon, 1999	Canada	Pilot study	Adult	12	CD	Active and remission	Walking	12 weeks, 3 times/week for 20–35 min	QoL, disease activity score, VO_2_peak
Ng, 2007	Canada	Randomized prospective study	Adult	16	CD	Remission	Walking	12 weeks, 3 times/week for 30 min	QoL, disease activity score
Robinson, 1998	UK	RCT	Adult	60	CD	Remission	Home‐based, low‐impact exercises	52 weeks, 2 times/week	Disease activity score, BMD
Seeger, 2020	Germany	Pilot three‐arm parallel group RCT	Adult	22	CD	Remission	Endurance/muscle training	12 weeks, 3 times/week for 30–40 min	QoL, disease activity score, faecal calprotectin, HGS
Tew, 2019	UK	Pilot RCT	Adult	25	CD	Remission	High intensity/moderate intensity interval training	12 weeks, 3 times/week for 28–38 min	QoL, disease activity score, faecal calprotectin, VO_2_peak
van Erp, 2021	The Netherlands	Pilot study	Adult	25	CD, UC and IBD‐U	Remission	Personalized exercise programme with aerobic training and progressive resistance training	12 weeks, 3 times/week for 60 min	QoL, BFM, VO_2_peak
Zhao, 2022	China	Randomized, double‐blind, placebo‐controlled trial	Adult	13	N/A	Active and remission	Resistance training	8 weeks, 3 times/week	CRP, ESR, SMM, HGS
de Souza, 2014	Brazil	Abstract	Adult	19	CD and UC	N/A	Quadriceps progressive resistance training	8 weeks, 2 times/week for 20 min	QoL, quadriceps strength
Fagan, 2019	New Zealand	Abstract	Adult	59	CD and UC	N/A	N/A	17 weeks, 5 times/week for 10 min	QoL, disease activity score
Fischbach, 2009	Germany	Abstract	Adult	30	CD	N/A	N/A	12 weeks, 2 times/week for 90 min	QoL, disease activity score

Abbreviations: BFM = body fat mass; BMD = bone mineral density; CD = Crohn's disease; CRP = C‐reactive protein; ESR = erythrocyte sedimentation rate; HGS = handgrip strength; IBD‐U = inflammatory bowel disease unclassified; LBM = lean body mass; QoL = quality of life; SMM = skeletal muscle mass; UC = ulcerative colitis.

### 3.3 Improvement of Disease‐Specific HRQoL

**FIGURE 2:**
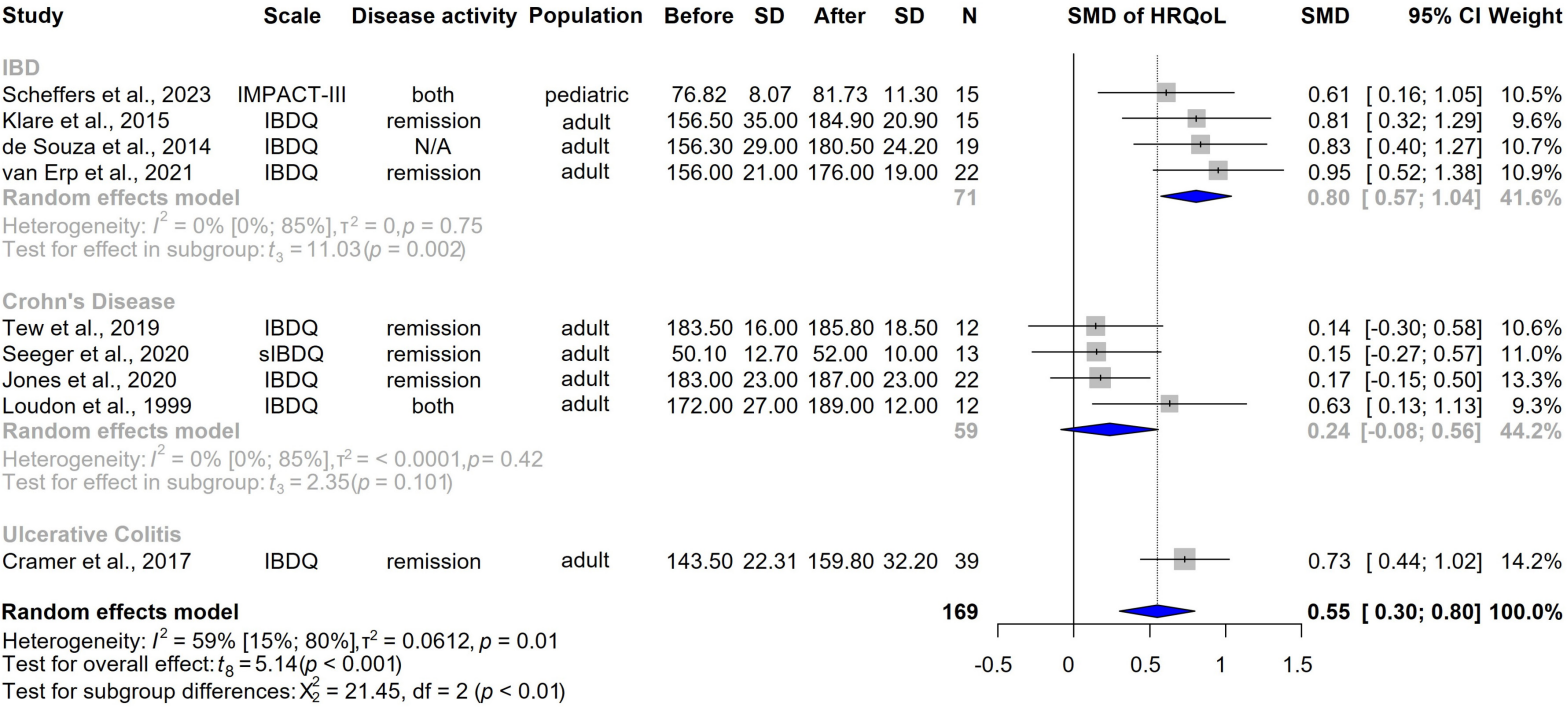
Impact of physical exercise on disease‐specific health‐related quality of life. CI = confidence interval; HRQoL = health‐related quality of life; IBD = inflammatory bowel disease; IBDQ = inflammatory bowel disease questionnaire; *N* = number of patients; SD = standard deviation; sIBDQ = short version of inflammatory bowel disease questionnaire; SMD = standardized mean difference.

Eight studies [26, 28, 29, 32, 34, 38, 43, 45] reported data using the disease‐specific IBDQ [47], and one [33] used the short version of the IBDQ [48]. Furthermore, another study [32] utilized the disease‐specific IMPACT‐III [49] developed for children. Data from 169 patients revealed a statistically significant improvement in QoL following interventions (SMD 0.55 [CI, 0.30–0.80, *I* ^2^ = 59%, CI, 15%–80%, *p* < 0.001]) (Figure 2).

One study that used the IBDQ could not be included due to missing data [39]. However, Fagan et al. reported that 31% of participants experienced clinically meaningful improvement. All studies applying general HRQoL instruments utilized the SF‐36 [27, 46] and SF‐12 [40] for QoL data and showed significant [27] or non‐significant [40, 46] improvements (*p* < 0.05).

All subscale analyses of the bowel, emotional, social, and systemic subscales showed an increase in HRQoL (Figure S1).

### 3.4 Change in Disease Activity Score

**FIGURE 3:**
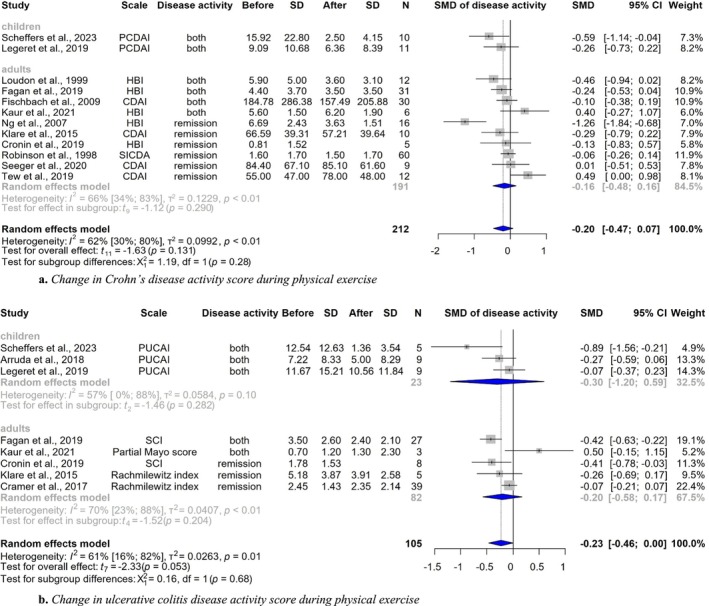
(a) Change in Crohn's disease activity score during physical exercise. (b) Change in ulcerative colitis disease activity score during physical exercise. CDAI = Crohn's disease activity index; CI = confidence interval; *N* = number of patients; PCDAI = paediatric Crohn's disease activity index; HBI = Harvey–Bradshaw Index; SD = standard deviation; SMD = standardized mean difference.

Fourteen studies assessed disease activity scores [26, 27, 29, 30, 31, 32, 33, 34, 36, 39, 40, 42, 43, 46], involving 212 patients with CD and 105 patients with UC. To measure CD activity, we included studies that used the CDAI [50], Harvey Bradshaw Index/Simple Index of Crohn's Disease Activity [51] and PCDAI [52]. For UC activity, we included studies that used the Rachmilewitz Index [53], Simple Colitis Index [54], Partial Mayo Score [55] and PUCAI [56]. The effect size showed a tendency for improvement both in the CD and UC group (CD: SMD: −0.20 [CI, −0.47 to 0.07, *I* ^2^ = 62%, CI, 30%–80%, *p* = 0.131] and UC: SMD −0.23 [CI, −0.46 to 0.00, *I* ^2^ = 61%, CI, 16%–82%, *p* = 0.053]) (Figure 3a,b).

### 3.5 Impact of Physical Exercise on Levels of Inflammatory Markers

**FIGURE 4:**
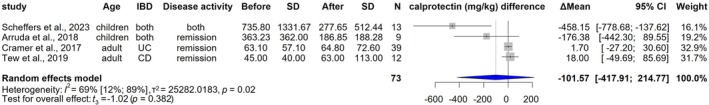
Shift in faecal calprotectin levels during structured physical exercise. CD = Crohn's disease; CI = confidence interval; IBD = inflammatory bowel disease; *N* = number of patients; SD = standard deviation; UC = ulcerative colitis; Δmean = mean difference.

Six studies [26, 27, 29, 32, 35, 42] reported alteration in CRP levels, and four reported ESR changes [26, 32, 35, 42]. The MD for both parameters decreased, though the difference was not statistically significant (CRP: MD −0.13 [CI, −0.30 to 0.03, *I* ^2^ = 47%, CI, 0%–79%, *p* = 0.096] and ESR: MD −6.21 [CI, −13.31 to 0.90, *I* ^2^ = 91%, CI, 80%–96%, *p* = 0.069]) (Figures S2 and S3). Faecal calprotectin was measured in six studies [26, 29, 32, 33, 34, 36, 42] and the MD was −101.57 (CI, −417.91 to 214.77, *I* ^2^ = 69%, CI, 12%–89%, *p* = 0.382) (Figure 4).

### 3.6 Changes in Muscle Strength

**FIGURE 5:**
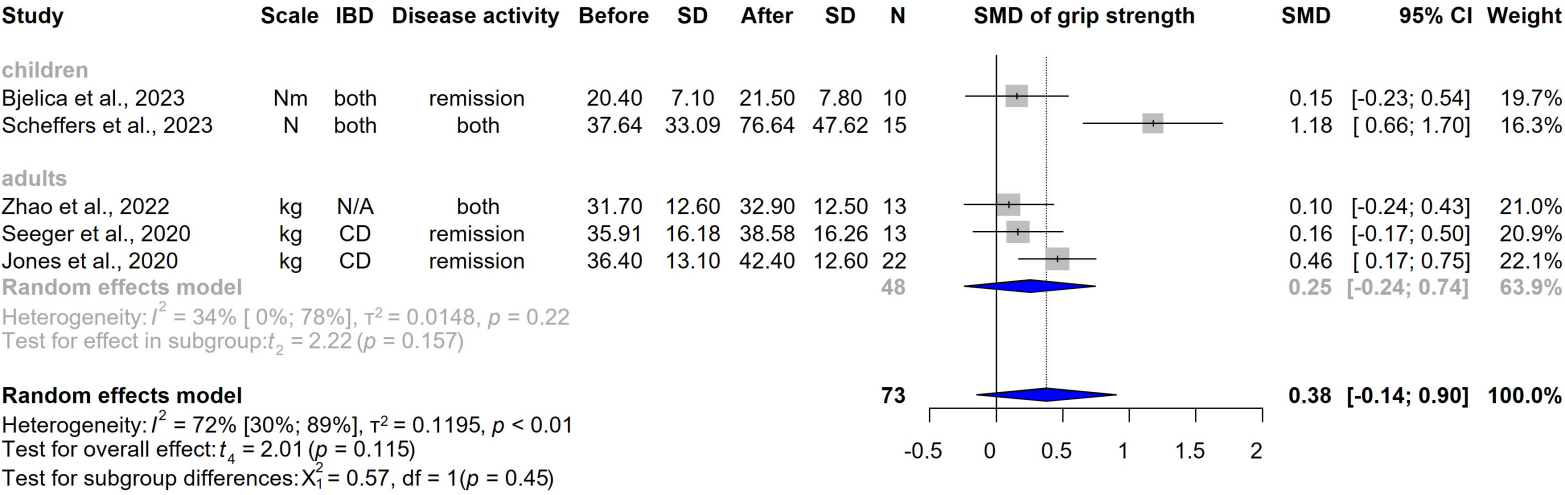
Impact of physical exercise on handgrip strength. CD = Crohn's disease; CI = confidence interval; IBD = inflammatory bowel disease; *N* = number of patients; SD = standard deviation; SMD = standardized mean difference.

HGS was measured in two paediatric [32, 37] and three adult studies [28, 33, 35]. Data from 73 patients showed a non‐significant increase (SMD: 0.38 [CI, −0.14 to 0.90, *I* ^2^ = 72%, CI, 30%–89%, *p* = 0.115]) (Figure 5). Quadriceps strength was reported in three studies [33, 37, 38] with an SMD of 0.75 (CI, −1.58 to 3.08, *I* ^2^ = 93%, CI, 84%–97%, *p* = 0.302) (Figure S4).

### 3.7 Improvement in Aerobic Fitness

VO_2_ measurements significantly improved based on the result of two studies involving children [32, 37] and three studies involving adults [34, 43, 45], including 70 patients (MD 1.88 [CI, 1.34–2.43, *I* ^2^ = 0%, CI, 0%–79%, *p* < 0.001]) (Figure S5).

### 3.8 Body Composition During Structured Physical Exercise: Qualitative Synthesis

Five studies evaluated BFM [27, 32, 37, 44, 45], by employing diverse methodologies for body composition assessment, including dual‐energy X‐ray absorptiometry and skinfold thickness measurements. Of the studies identified, two were conducted in adult populations, and three were conducted in paediatric populations. The results of two studies indicated an increase in BFM [32, 44], whereas the other three reported a decrease [27, 37, 45].

LBM was assessed in three studies [27, 37, 44]; despite the use of identical measurement tools, the data could not be analysed due to heterogeneous data presentation (*Z*‐scores, raw values and percentages). The results of the three studies indicate an improving trend as a result of physical intervention, although only two were statistically significant [27, 37].

BMD was reported in two paediatric and two adult studies [28, 31, 37, 44]. All four studies showed an improvement in BMD, although only one study [44] reported a significant increase.

SMM was measured in only one study [35], which found that height‐adjusted appendicular SMM significantly improved as the intervention progressed.

### 3.9 Change in Sedentary Time

Sedentary time was measured in only three studies [32, 37, 41]. Data, however, could not be pooled due to the different data reporting methods. Two studies [32, 37] showed a trend towards a reduction in sedentary time after the intervention. The third, however, did not [41].

### 3.10 The Role of Exercise Type

Participants in five studies engaged in aerobic exercises [29, 33, 34, 41, 42], whereas four studies focused on resistance training [28, 31, 33, 35]. In addition, five studies involved low‐intensity workouts [26, 30, 36, 40, 43]. Finally, four studies employed a combination of mixed exercises [27, 32, 37, 45], which included both aerobic and resistance training. We planned to compare each outcome by exercise type, but the data were not sufficient to perform an accurate analysis. However, the data did not suggest a difference in the QoL by exercise type (Figure S6).

### 3.11 Risk of Bias Assessment

The detailed results of the risk of bias assessment are presented in Table S3. Of the studies included, three had a low overall risk of bias [28, 32, 34], eight had a moderate risk [26, 27, 29, 33, 35, 36, 40, 41] and ten had a high risk [30, 31, 37, 38, 39, 42, 43, 44, 45, 46]. The most common reason for the moderate risk of bias was the absence of a prospective study size calculation. The high risk of bias was typically due to the same underlying issue, combined with a loss to follow‐up of more than 5%. Other causes of high risk included unclear definitions of inclusion/exclusion criteria and an inadequate follow‐up period (more than 12 weeks after initial measurement).

### 3.12 Publication Bias and Heterogeneity

Between‐study heterogeneity was described using Higgins and Thompson's *I* ^2^ statistics [57]. High heterogeneity in muscle strength and inflammatory markers (CRP, ESR and faecal calprotectin) is likely to arise from methodological and biological variability. In the case of muscle strength, differences in assessment methods, testing protocols, equipment and participant characteristics (e.g., age, sex, nutritional status and baseline activity levels) can influence the results obtained. Inflammatory markers are subject to natural fluctuations and variations in baseline disease activity. They are also affected by differences between IBD subtypes and the effects of concurrent medications, particularly corticosteroids or biologics. Further inconsistency is introduced by additional variability in laboratory assays and cut‐off values, which can potentially obscure the true impact of structured exercise on these outcomes.

Small study publication bias was assessed by visual inspection of funnel plots if there were at least 5 studies and Egger's test [58] if there were at least 10 studies. We considered possible small study bias if the *p* value was less than 0.1. The funnel plot could be assessed for HRQoL, CRP, aerobic fitness, HGS and disease activity scores and revealed publication bias in the case of HRQoL, HGS and disease activity scores (Figures S7–S11). The publication bias in these outcomes can be explained by several factors. Smaller trials of structured exercise for IBD have less control over confounding factors, and these trials/studies often end up reporting larger improvements, which are sometimes due to chance. Different HRQoL instruments, HGS protocols and disease activity scores can produce inconsistent results. Smaller or less rigorous studies may select methods that are more likely to demonstrate significant change, thereby inflating effect estimates.

## 4 Discussion

In this study, we summarized the association between structured physical exercise and objective and subjective outcomes in patients with IBD. Using the before–after approach, we were able to present the changes in the outcomes investigated over a few weeks of exercise. Our findings indicated a significant improvement in HRQoL, with the most substantial gains observed in the systemic subscale. This means the greatest improvements were in fatigue, energy levels, sleep quality and general health. Furthermore, muscle strength and aerobic fitness improved significantly. Although there was a trend towards improvement in disease activity scores and inflammatory markers (CRP, ESR and faecal calprotectin), these changes were not statistically significant. Although a meta‐analysis was not feasible for some outcomes, preliminary evidence suggests that exercise is associated with a decrease in BFM and an increase in LBM, BMD, SMM and sedentary time.

Similar to the findings of a previous meta‐analysis by Jones et al. [59], the results of our study indicated that HRQoL improved with regular physical exercise. The subscale analysis demonstrated that moderate improvements could be attained in both the systemic and emotional subscales, even though 75% of the patients were in remission or had mildly active disease with relatively high HRQoL at the beginning of some studies.

Disease activity scores non‐significantly decreased in patients with CD and UC. This lack of significance is likely due to the limited potential for improvement. More than half of the analysed studies excluded patients with active disease; as a result, baseline activity scores were low with less likelihood to improve. Furthermore, many of the studies that included patients with active disease did not report separately the data of participants with active and inactive disease. Consequently, a subgroup analysis based on disease activity was not possible.

Our analysis showed that inflammatory markers decreased during the intervention, although the difference was not significant. We observed more pronounced changes in studies that included patients with active disease. However, it is essential to note that CRP levels were only slightly elevated in one study [42], and ESR levels were within the normal range in all four studies [26, 32, 35, 42]. These findings are consistent with previous reviews [59, 60]. This trend may reflect a modest anti‐inflammatory effect of structured exercise, which larger and longer trials may be better positioned to detect. We also acknowledge that variation in exercise protocols, particularly in terms of intensity, duration and type of exercise, may influence the responsiveness of these markers. For example, higher‐intensity or longer‐duration interventions may produce greater systemic and mucosal anti‐inflammatory effects, whereas shorter or lower‐intensity programmes may be insufficient to generate measurable changes in these biomarkers [61]. Future trials would also benefit from including participants with moderate or severe active disease to better assess the impact of interventions on disease activity and inflammatory markers.

We also planned to perform subgroup analysis by age group, disease activity, intervention type and diagnosis. However, data were not sufficient for these analyses. Only a few paediatric studies assessed these outcomes, and many of the included studies, even those involving the adult population, did not report the data separately based on the planned subgroups (active and inactive patients, or patients with CD and UC). It is noteworthy that the paediatric studies showed similar changes in all outcomes compared with adults (Figure 3b). Most studies included patients in remission or with mild activity and did not report specific results based on activity. Results were similar in studies with patients in remission and mild disease activity. Disease activity and inflammatory markers did not worsen. Additionally, the majority of studies reported no relapses during the intervention period, suggesting that the physical intervention did not cause disease flare‐ups. This has important implications for clinical practice: patients with mild disease may also benefit from exercise. The optimal type of exercise for patients with IBD remains uncertain, as variability in the type and duration of interventions prevented a definitive analysis. Nonetheless, all interventions showed positive effects across outcomes, with none leading to a decline. Although it is still unclear which form of physical activity is most effective in improving the well‐being of patients with IBD, existing evidence indicates that all interventions have been associated with some level of benefit.

### 4.1 Strengths and Limitations

One of the strengths of our analysis is the comparison of changes to baseline measurements, which demonstrates the benefits of exercise at the individual patient level. In addition, we analysed patients with IBD based on their disease activity separately. Furthermore, a comprehensive analysis of objective and subjective parameters associated with the beneficial effects of physical activity was conducted. Due to the heterogeneity of the interventions, including variations in duration, type, supervision and co‐interventions (e.g., dietary), the analysis was further complicated by the fact that the outcomes were reported in various units of measurement. Additionally, due to the limited number of studies on the paediatric population, it was not feasible to analyse the data separately by age group. Alongside the aforementioned factors, most exercise interventions were of relatively short duration, and several studies excluded patients with active disease. These factors may limit our ability to detect sustained effects and reduce the generalizability of our findings to patients with more severe disease activity.

### 4.2 Implications for Practice and Research

Our results suggest that physicians should encourage patients with IBD, irrespective of whether they are in remission or have mild disease activity, to engage in regular physical activity. This may include walking, cycling or resistance training, which should be tailored to the fitness level and disease state of the patient. Physical activity should be incorporated into a broader, multidisciplinary guideline that also addresses nutrition, mental health support and regular medical evaluations.

We recommend that future studies include patients with active disease and increase the focus on the paediatric population, as IBD is a lifelong condition, with 25% of patients diagnosed in childhood [2]. We also advise researchers to provide detailed descriptions of interventions and report outcomes using commonly accepted units of measurement. Our findings suggest that the next step in physical activity research on patients with IBD should be to explore the most beneficial forms of exercise for this population.

## 5 Conclusions

Our results suggest that regular physical activity in patients with IBD significantly improves HRQoL and overall physical condition. Furthermore, disease activity scores and inflammatory markers tend to decrease, even among patients in remission. It was not possible to conduct a statistical analysis to ascertain the effect of exercise on body composition. However, the extant data suggest that exercise exerts a positive effect on these parameters. Based on our findings, patients with IBD in remission and mild activity should be encouraged to engage in regular physical activity. Further research is required to identify the most beneficial forms of exercise for this population.

## Funding

Funding was provided by the János Bolyai Research Scholarship of the Hungarian Academy of Sciences (BO/00693/25/5), by the ÚNKP‐23‐5‐SE22 New National Excellence Program of the Ministry for Culture and Innovation from the source of the National Research, Development and Innovation Fund and by the 2024‐2.1.2‐EKÖP‐KDP New National Excellence Program of the Ministry for Culture and Innovation from the source of the National Research, Development and Innovation Fund.

Sponsors had no role in the design, data collection, analysis, interpretation and manuscript preparation.

## Ethics Statement

No ethical approval was required for this systematic review with meta‐analysis, as all data were already published in peer‐reviewed journals. None of the patients were involved in the design, conduct or interpretation of our study. The datasets used in this study can be found in the full‐text articles included in the systematic review and meta‐analysis.

## Conflicts of Interest

The authors declare no conflicts of interest.

## Supporting information


**Table S1:** PRISMA Checklist 2020.
**Table S2:** Search key of the systematic literature.
**Table S3:** Risk of bias assessment with the MINORS tool.
**Figure S1:** Improvement of disease‐specific health‐related quality of life subscales.
**Figure S2:** Impact of physical exercise on the levels of CRP.
**Figure S3:** Impact of physical exercise on the levels of ESR.
**Figure S4:** Change in quadriceps strength.
**Figure S5:** Improvement of aerobic fitness.
**Figure S6:** Change in disease‐specific health‐related quality of life with exercise subgroup analysis.
**Figure S7:** Funnel‐plot for disease specific health related quality of life.
**Figure S8a:** Funnel‐plot for Crohn's disease activity score.
**Figure S8b:** Funnel‐plot for ulcerative colitis activity score.
**Figure S9:** Funnel‐plot for C‐reactive protein.
**Figure S10:** Funnel‐plot for handgrip strength.
**Figure S11:** Funnel‐plot for aerobic fitness

## Data Availability

All data relevant to the study are included in the study or have been uploaded as supplementary information available online. The data supporting this systematic review and meta‐analysis are drawn from studies previously published in the literature and referenced accordingly. The processed data are available in the paper and the Supporting information S1.
